# A UNIQUE FINDING – MELANOSIS OF THE BLADDER

**DOI:** 10.51894/001c.123134

**Published:** 2024-08-31

**Authors:** Lauren Nesi

**Affiliations:** 1 Urology Detroit Medical Center

## 99

### INTRODUCTION AND OBJECTIVES

Melanosis of the bladder is a rare and benign condition. There have been approximately 30 case reports described in the literature. In this article, we present a 72-year-old male undergoing workup for gross hematuria, lower urinary tract symptoms and recurrent infections found to have melanosis cystica.

### CASE DESCRIPTION

Patient is a 72-year-old male with a history of gross hematuria, recurrent UTIs and enlarged prostate with lower urinary tract symptoms. Due to his hematuria, patient was taken to the operating room for cystoscopy with possible bladder biopsy. Under cystoscopic examination, the patient was found to have two 1cm bladder stones and an abnormal darkening of the bladder wall. The bladder mucosa was found to be starkly abnormal, appearing black and velvety. This hyperpigmentation was seen diffusely, but most notably seen on the posterior bladder wall. No other lesions were identified. Biopsies were obtained from darkly pigmented areas. Cystolitholapaxy was also performed. Patient returned to the clinic approximately one month later, reporting resolution of symptoms with no further gross hematuria or voiding dysfunction. Two samples were sent for analysis. The biopsy revealed urothelium with intracytoplasmic dark pigments negative for Periodic Acid Schiff, Human Melanosome 45, SOX10, p53 and Gomori’s iron stain. The pigments were positive for Fontana stain, consistent with melanin. The pigmentation also disappeared after treatment with bleach.There was no evidence of carcinoma. These findings are pathognomonic for benign melanosis.

### CONCLUSION

Melanosis of the bladder is an extremely rare finding characterized by urothelial cell hyperpigmentation associated with abnormal increased melanin deposition in cells. Past case reports show an association with a number of different symptoms including dysuria, urinary frequency, hematuria, incontinence and retention. No reported cases are described with concurrent bladder malignancy. The benign nature of this condition contrasts with its concerning presentation, questioning the need histopathological confirmation. There is a paucity of data to provide any recommendations regarding regular surveillance. Hence, melanosis cystica, though rare, should be considered in the differential diagnosis of urothelial hyperpigmentation. Future research is warranted to better understand long-term prognostic outcomes and develop a standardized approach for management.

### PROGRAM PLANNING COMMITTEE

C. Patricia Obando S., PhD.

Associate Dean and DIO, Graduate Medical Education

Associate Professor- MSU College of Osteopathic Medicine- Statewide Campus System

John L. Goudreau, DO, PhD, FACN

Associate Dean, Research Co-Director of the DO/PhD program- College of Osteopathic Medicine

Associate Professor, Department of Neurology

Director, Clinical & Translational Science Institute

Brian C. Schutte, PhD

Associate Professor of Microbiology and Molecular Genetics and of Pediatrics

Co-director for DO/PhD Program- MSU College of Osteopathic Medicine 

Colleges of Osteopathic Medicine, Natural Sciences and Human Medicine

Rana Ismail, PhD, MSc, CPHQ

Director of Research

Editor, Spartan Medical Research Journal (SMRJ)

MSU College of Osteopathic Medicine- Statewide Campus System

Carolina Restini, PharmD, PhD

Associate Professor

Michigan State University College of Osteopathic Medicine, MUC, and DMC

Department of Pharmacology & Toxicology

Furqan B. Irfan, MBBS (MD), PhD, MBA-candidate

Associate Professor, Department of Neurology

Director Research Development, College of Osteopathic Medicine

Institute of Global Health, Michigan State University

Yasser Aldhamen, MSc, PhD, MBA

Assistant Professor

Microbiology and Molecular Genetics Department- Michigan State University.

